# Breaking Patterns of Environmentally Influenced Disease for Health Risk Reduction: Immune Perspectives

**DOI:** 10.1289/ehp.1001971

**Published:** 2010-05-18

**Authors:** Rodney R. Dietert, Jamie C. DeWitt, Dori R. Germolec, Judith T. Zelikoff

**Affiliations:** 1 Department of Microbiology and Immunology, College of Veterinary Medicine, Cornell University, Ithaca, New York, USA; 2 Department of Pharmacology and Toxicology, Brody School of Medicine, East Carolina University, Greenville, North Carolina, USA; 3 National Toxicology Program, National Institute of Environmental Health Sciences, National Institutes of Health, Department of Health and Human Services, Research Triangle Park, North Carolina, USA; 4 Nelson Institute of Environmental Medicine, New York University Langone School of Medicine, Tuxedo, New York, USA

**Keywords:** asthma, developmental immunotoxicity, health risks, immune dysfunction, inflammation, intervention, metabolic syndrome, patterns of disease, prevention, safety testing

## Abstract

**Background:**

Diseases rarely, if ever, occur in isolation. Instead, most represent part of a more complex web or “pattern” of conditions that are connected via underlying biological mechanisms and processes, emerge across a lifetime, and have been identified with the aid of large medical databases.

**Objective:**

We have described how an understanding of patterns of disease may be used to develop new strategies for reducing the prevalence and risk of major immune-based illnesses and diseases influenced by environmental stimuli.

**Findings:**

Examples of recently defined patterns of diseases that begin in childhood include not only metabolic syndrome, with its characteristics of inflammatory dysregulation, but also allergic, autoimmune, recurrent infection, and other inflammatory patterns of disease. The recent identification of major immune-based disease patterns beginning in childhood suggests that the immune system may play an even more important role in determining health status and health care needs across a lifetime than was previously understood.

**Conclusions:**

Focusing on patterns of disease, as opposed to individual conditions, offers two important venues for environmental health risk reduction. First, prevention of developmental immunotoxicity and pediatric immune dysfunction can be used to act against multiple diseases. Second, pattern-based treatment of entryway diseases can be tailored with the aim of disrupting the entire disease pattern and reducing the risk of later-life illnesses connected to underlying immune dysfunction. Disease-pattern–based evaluation, prevention, and treatment will require a change from the current approach for both immune safety testing and pediatric disease management.

During the past 30 years, many environmental health concerns linked with early-life exposures to environmental agents have centered on diseases that are increasing in prevalence in many countries, are often chronic in nature, and can significantly affect both health care needs and quality of life. Among those diseases and conditions are childhood asthma and other allergic diseases, various autoimmune conditions (e.g., type 1 diabetes, arthritis, autoimmune thyroiditis, celiac disease, multiple sclerosis), neurological tissue disorders (e.g., autism, schizophrenia, Parkinson’s disease, Alzheimer’s disease), and metabolic and cardiovascular disorders (e.g., childhood obesity, cardiac disease, atherosclerosis). Most of these diseases have at least three features in common: early-life exposures to environmental risk factors (i.e., chemical agents or pathogens) are thought to be important in disease risk; immune-inflammatory insult or dysfunction is evident; and the disease itself and/or early biomarkers of later-life disease are prominent in previously exposed children.

Childhood immune-based diseases with environmental risk factors can affect as much as 25% of the pediatric population in some developed countries (Dietert and Zelikoff 2009). Although this impact of environmentally influenced, immune-based disease is of significant concern, it does not reflect the full extent of the problem between environmental risk factors and immune system health. Among subpopulations of children, significant illnesses such as asthma and type 1 diabetes are not only the end result of environmental exposures interacting with genetic background; they are the entryway into even larger environmentally associated health concerns. This is because asthma, type 1 diabetes, and many other illnesses that are prominent in children are interlinked to an elevated risk of other diseases via a superstructure that has been termed a “pattern” of disease (Dietert and Zelikoff 2010).

At the heart of a disease pattern is the first-onset disease or condition. The earliest disease has been termed the “entryway” disease for a specific pattern (Dietert and Zelikoff 2010). Once that disease arises, elevated health risks for comorbidities are often triggered in the diagnosed pediatric population. These secondary diseases may occur during childhood or later in life. Although patterns of diseases are most often connected via underlying biological mechanisms, in some cases a predominant course of medical treatment may have the potential to contribute to an elevated risk for certain secondary conditions.

The contributions of immune dysfunction and immune-based childhood illnesses and diseases to larger “patterns” of disease are only beginning to be fully appreciated. This latter point is particularly significant when environmental risk factors are viewed across a lifetime. If a diagnosis of asthma in childhood implies elevated health risks for several other serious adult-onset diseases, then there is an added value in preventing childhood asthma if such action might reduce the risk of additional health conditions later in life. In fact, effective immune homeostasis is a crucial aspect of health across all age groups. Even in the latest stages of life, disruption of immune homeostasis appears to differentiate dementia from normal cognitive function (Katsel et al. 2009).

In the present review, we illustrate several disease patterns where immune and inflammatory dysfunction appear to be most important. We show how the intersection of disease patterns can shed light on potential multiple routes for developing a single disease or condition. Finally, we describe how a pattern of diseases can be substituted for the traditional single-disease focus to optimize integrative environmental health protection strategies.

## Underestimating the Role of Environmentally Influenced Immune Dysfunction in Disease

Historically, the immune system has been viewed first and foremost in the context of primary immune organs (e.g., thymus) and secondary sites of acquired immune responses (e.g., spleen and lymph nodes). This has been strengthened by the focus on host defense against external assaults (often microbiological) that were critical in defining an important role of the immune system. Additional events involving immune cells in other tissues and organs were largely defined by the nature and physiological function of that tissue (e.g., cardiovascular, neurological, reproductive, endocrine, hepatic, respiratory). This resulted in many immune-based diseases being categorized and treated by medical specialists based on tissue location and biological function rather than by the underlying cause of the disease (Dietert, in press). As will be discussed in the context of breaking or disrupting a pattern of disease, the focus on disease location (by system) rather than on the underlying cause may actually hamper efforts to break the cycle of diseases influenced by environmental risk factors across a lifetime.

Viewing the immune system in a manner restricted to primary and secondary lymphoid organs overlooks additional immune functions that are equally important for health and well-being. Early in life, immune cells are distributed to virtually every tissue and organ of the body, where they serve as sentries for organ integrity and homeostatic regulators of the tissue’s physiological function. Most of these cells either are macrophage-lineage related or represent highly specialized lymphocyte populations (e.g., intraepithelial lymphocytes). In fact, in organs critical for metabolism and growth (e.g., liver), the functional status of the majority of the cell populations (hepatocytes) can be significantly influenced, if not largely dictated, by the minority resident immune cell population: the Kupffer cells (Neyrinck et al. 2009; Usynin and Panin 2008; Wang et al. 2009).

Macrophage-like cells exhibit unique phenotypes and differential environmental sensitivity depending on their location in the body. As a result, Kupffer cells in liver, Langerhans cells and keratinocytes in skin, alveolar macrophages in lung, microglia and astrocytes in brain, foam cells of atherosclerotic plaques, preadipocytes in adipose tissue, osteoclasts in bone, and testicular macrophages, although all related in lineage, exhibit significant differences in terms of either specific leukocyte function or environmental sensitivity. Dysfunction among these resident macrophage populations can cause diseases *in situ* that, as mentioned, are usually labeled and medically treated based on the involved tissue. But, if the actual environmental exposures involve earlier-in-life induction of immune dysfunction, tissue-specialized treatments may only address immune-mediated loss of that tissue’s physiological function and may not correct the underlying immune dysfunction. The danger across a lifetime is that the unaddressed dysfunction may elevate additional health risks associated with aging and exposure to additional environmental triggers. Not surprisingly, the immune cells in question (i.e., macrophages) are particularly susceptible to early-life modulation and imprinting by environmental risk factors in ways that can affect even basic growth parameters across a life course (DeWitt and Dietert 2010).

Examples of diseases and conditions that have an established immune dysfunction as a basis but that are often categorized by tissue or organ pathology are atherosclerosis (cardiovascular), asthma (respiratory), celiac disease (gastrointestinal), psoriasis (dermal), and multiple sclerosis (neurological) (Dietert, in press). For other illnesses where immune dysfunction or immune insult is suspected as a possible pathway to disease, recognition of likely immune involvement is even less apparent (e.g., neurobehavioral, neurodeficit, and fatigue disorders).

## Environmental Disruption of the Developing Immune System

The developing immune system is highly susceptible to modification and disruption by environmental influences and is at significantly greater risk than the fully matured immune system of an adult (Luebke et al. 2006). The health risks and concerns for the developing immune system are real, and specific examples of human vulnerability are known (Selgrade 2007). In part, the elevated risk of developmental immune dysfunction is based on the fact that numerous unique immune maturational events are programmed into specific windows of prenatal and neonatal development. These maturational events represent building blocks upon which subsequent changes in the immune system are built.

The period in which each unique immune maturation event occurs is known as a “critical window of immune vulnerability” (Dietert et al. 2000). For example, during thymocyte maturation in the thymus, negative selection occurs to eliminate thymocytes that have autoreactive capabilities and that are likely to promote later-life autoimmunity. Specific environmental agents such as lead, corticosteroids, and halogenated hydrocarbons are known to disrupt one or more specific developmental windows of immune vulnerability. Critical windows for the developing immune system were originally described a decade ago (Dietert et al. 2000; Landreth 2002) and were later updated to include additional events susceptible to disruption by environmental risk factors (Dietert and Piepenbrink 2006). More recently, the critical windows were subdivided into systemic versus local events that were tailored for disease-specific health risks (Dietert and Dietert 2008). Later in this review, we extend this concept to suggest potential critical windows of immune development for a prototypic pattern of diseases and conditions known as metabolic syndrome.

## Adverse Outcomes of Developmental Immunotoxicity

Developmental immunotoxicity (DIT) can be defined as adverse effects on the immune system resulting from exposure to environmental risk factors, including chemical, biological, physical, or physiological factors, before adulthood (Luster et al., in press). With most patterns of disease, it is exposure to environmental risk factors prior to adulthood that is of greatest concern. Even for diseases with adult or geriatric onset, the underlying disease mechanisms are usually established much earlier in life, and biomarkers of increased risk of future disease usually appear well in advance of disease onset (Kwiterovich 2008). This is certainly true for diseases with immune linkages.

**Figure 1:**
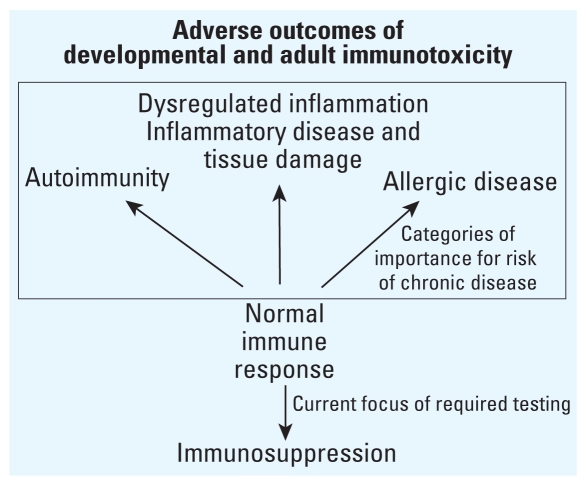
The adverse outcomes of developmental and adult immunotoxicity. The detection of immune suppression is useful, but it is the remaining adverse immune outcomes (boxed) that appear to be the more important public health risks when one considers prevalence of chronic disease and significant patterns of immune-based disease.

Adverse outcomes of DIT can take many forms as shown in Figure 1. These include not only immunosuppression, but also increased risk of allergic and autoimmune diseases, inflammatory and metabolic dysfunction, and health problems associated with immune-inflicted damage of tissues and organs (Dietert 2009b). Ironically, the historic and current focus of both DIT and adult immunotoxicity testing is on immunosuppression and potential allergenicity of test compounds (Dietert and DeWitt 2010). Although detection of immune suppression is useful, it is the remaining adverse immune outcomes (Figure 1, box) that appear to be more important public health risks when one considers prevalence of chronic disease and significant patterns of immune-based disease (Dietert and Zelikoff 2009). One goal is to better align immunotoxicity testing with prioritized health risk reduction. As will become evident in the subsequent discussion of patterns of disease, much of the concern for immune-based chronic disease occurs less with issues of immunosuppression (or a single chemical’s allergenicity) than with the other types of adverse outcomes of immunotoxicity (i.e., predisposition for autoimmunity, allergy, inflammatory disease, and inflammation-related tissue damage).

One of the most significant public health concerns of today is influenza. Risk of complications and even death from influenza are among the major historic and present-day concerns. Given that influenza virus infection is the initiator of the disease, one might easily assume that immunosuppression is the greatest DIT risk relative to later-life influenza. But this assumption is less obvious than it might seem. Inappropriate immune responses are at least as important in determining life-shortening or life-threatening complications from influenza as are insufficient levels of immune responses (Baskin et al. 2009; Cillóniz, et al. 2009; Hussell and Cavanagh 2009; Lin et al. 2008). Problematic immune and inflammatory responses to the initial viral infection in the airways can result in secondary bacterial infections (Rothberg et al. 2008) and severe damage to epithelial layers (Hussell and Cavanagh 2009), which can increase the risk of mortality.

Additionally, exposure to infectious agents like the influenza virus can trigger a wide range of diseases when preexisting and underlying immune dysfunctions are present (Dietert 2009c). The nature of the disease outcome appears to depend upon the nature of the underlying immune dysfunction and the type of infectious agent encountered. This type of relationship has been suggested in the case of sudden infant death syndrome (Blood-Siegfried et al. 2002, 2008). Additionally, infectious agent triggers of immune-dysfunction–based disease are thought to play a role in specific allergic, autoimmune, and inflammatory diseases as well as in some forms of childhood leukemia (Dietert 2009a; Greaves 2006).

Early-life exposure to environmental risk factors can significantly affect not only whether a childhood or adult immune response is adequate, but also whether an immune response is misdirected (i.e., prolonged, not long enough, or skewed toward inappropriate response types). Hogaboam et al. (2008) demonstrated that in mice a single *in utero* exposure to 2,3,7,8-tetrachlorodibenzo-*p*-dioxin (TCDD) resulted in production of a hyper- or misdirected inflammatory response in the lungs of the offspring that increased lung damage and mortality after later infection with influenza. The potential for inappropriate inflammation already existed in these mice as a result of prenatal exposure to TCDD but was not revealed or apparent until the animals were faced with a viral challenge later in life. Without proper and relevant challenges among DIT testing paradigms, relationships between early-life exposures and later-life responses to other environmental risk factors may be missed.

The questions of how, when, and in what way DIT testing should be conducted relates directly to current public health concerns in humans (Dietert and DeWitt 2010). Employing the full range of DIT testing relevant to our most significant public health concerns would increase the likelihood of reducing environmentally influenced health risks and associated disease prevalence for priority diseases, regardless of whether they are considered individually or as lifelong patterns of disease. For this reason, we support required DIT safety testing of drugs and chemicals.

## The Importance of Disease Patterns

Historically, environmentally influenced health risk prioritization, funding, research, and public health policies have been driven by a focus on individual diseases and conditions. Individual diseases usually take priority for public attention, investigation, and action. For this reason, diseases and conditions with documented environmental influences such as arthritis; asthma and allergies; breast, lung, and prostate cancer; heart disease; hearing loss; obesity; otitis media; periodontal disease; sleep disorders; and thyroid disorders are important categories of our public health effort. The benefit of the single-disease approach is that the research path from scientific understanding to public health and policy changes is reasonably direct and relatively transparent. But the downside is that the immediate return on this coordinated effort is likely restricted to the single disease in question, the known environmental risk factors that can be most readily addressed for that disease, and health management that is restricted to a single disease in isolation.

The potential problem with this historic approach is that preventing and managing environmentally influenced diseases can become little more than a series of uncoordinated piecemeal opportunities driven by a single-disease mantra. But diseases rarely occur in isolation. Instead, they exist as part of a pattern or superstructure of health risks across a lifetime (Dietert and Zelikoff 2010). With recent increased use of information contained in large medical databases, it is apparent that disease risk is organized into complex webs or patterns. The conditions that give rise to a pediatric disease often foretell the predominant health risks that will be faced in each subsequent decade of life (Dietert and Zelikoff 2009). With this in mind, the debate then becomes whether it is better to focus on single diseases or on landscapes (i.e., the webs) defined by multiple diseases and interconnected health risks. Therefore, it is worthwhile to consider the cost-effectiveness of protecting against single diseases versus patterns of diseases. In the following sections, we consider this point using recent, relevant examples.

## Metabolic Syndrome as a Pattern of Environmentally Influenced Disease

Metabolic syndrome is by definition a pattern of conditions and elevated disease risks (Grundy 2008). It is also a syndrome that features one type of immune dysfunction, excessive and misdirected immune inflammation, among its major characteristics. In fact, we would argue that the same approach that has led to heightened awareness, utility, and effectiveness of addressing the cluster of significant health risks connected to the metabolic syndrome pattern should be applied to a wider range of diseases and conditions. This approach is precisely what should be extended to other patterns of disease and used as the rule for reducing environmental health risks rather than as a novel exception.

Although some definitions differ slightly for what constitutes metabolic syndrome, it is generally thought of as the coexistence of three or more of the following five criteria: insulin resistance, high blood pressure, obesity, elevated triglycerides, and reduced high-density lipoprotein cholesterol (Grundy et al. 2004). Additionally, inflammatory dysfunction is a hallmark of metabolic syndrome (Fessler et al. 2009; Hevener et al., in press; Solomon et al. 2009; Suzuki et al. 2008). Indeed, underlying excessive and misdirected inflammation is thought to promote many of the diseases associated with metabolic syndrome (Milner et al. 2009; Rizvi 2009). The presence of metabolic syndrome is associated with an elevated risk of diabetes (Duvnjak and Duvnjak 2009), hepatic steatosis (Watanabe et al. 2008), kidney disease (Fakhrzadeh et al. 2009), stroke (Niwa et al. 2010), heart disease (Noda et al. 2009), and other cardiovascular morbidities, such as atherosclerosis (Kawamoto et al. 2007). Unlike other patterns of disease where immune dysfunction is clearly the underlying cause of the pattern, determining the precise relationship between metabolic syndrome and immune-inflammatory dysfunction requires additional study. The dysfunction here may be either causative of other metabolic syndrome conditions or, alternatively, associated with other metabolic syndrome conditions via some common mechanism.

### Metabolic syndrome as a prototype for pattern-based health risk reduction

**Figure 2:**
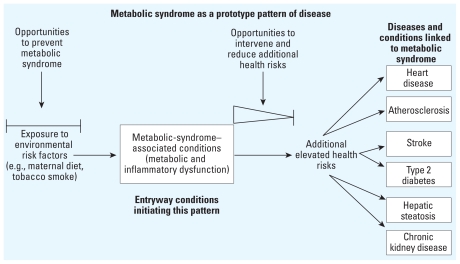
Prototype pattern of disease for health risk reduction connected to metabolic syndrome. Environmental exposures elevating the risk of metabolic syndrome are on the far left. Diseases and conditions with elevated risk associated with metabolic syndrome are on the far right. The first window on the left illustrates the opportunity to prevent initiation of metabolic syndrome by identifying and protecting against problematic environmental exposures that increase the risk of metabolic syndrome. The second window shows the opportunity to intervene in the metabolic syndrome pattern and reduce the risk for progression to associated diseases. Prompt intervention focused on the entire metabolic syndrome disease pattern may be more effective than treatment begun much later in life (as indicated by the diminishing triangle). Both the prevention and intervention strategies are important components of overall health risk reduction. The focus on patterns of disease deviates from the traditional single-disease approach.

Figure 2 illustrates the health risk pattern connected to metabolic syndrome, beginning with environmental risk factors and ending with elevated risk for several chronic diseases. As is discussed for subsequent patterns using pediatric immune-dysfunction–based entryway diseases, there are two major opportunities to reduce the impact of environmental risk factors on pattern-associated health risks and essentially prevent or disrupt the pattern (Figure 2). The first involves preventing the underlying cause through identification and avoidance of environmental risk factors that contribute to metabolic syndrome. The second opportunity involves intervening in the progression of the disease pattern (using medical treatment to address the overall pattern rather than just the initial presenting condition or disease). This would mean managing childhood or adult metabolic syndrome to minimize the risk of the associated diseases such as atherosclerosis, type 2 diabetes, and heart disease. As we will show with other immune-dysfunction–based patterns of disease, those same two opportunities exist once the pattern has been identified and can be used for health risk reduction.

### Inflammatory dysfunction and metabolic syndrome

**Figure 3:**
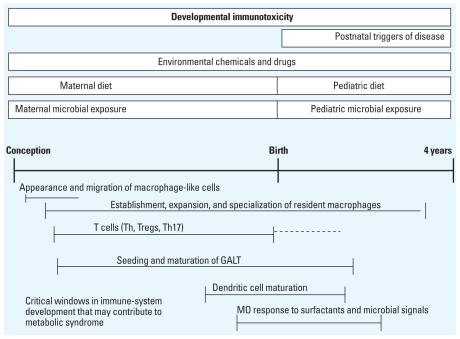
Early-life risk factors for metabolic syndrome: a timeline of human development from conception to 4 years of age. Abbreviations: GALT, gut-associated lymphoid tissue; MO, macrophage. Categories of different risk factors for metabolic syndrome are shown across the top. Below the timeline, windows of immune maturation are indicated that appear to be most relevant to environmental risk of metabolic syndrome. Combined, these factors and the critical windows are useful to further investigate the connection of an immune dysfunction–metabolic syndrome in early life. The dashed line indicates that immune maturation may continue after birth. Information derived from Dietert and Zelikoff (2008), Dietert and Dietert (2010), and DeWitt and Dietert (2010).

Several important points can be made about the metabolic syndrome pattern and immune dysfunction. Both can be established early in life, and biomarkers for metabolic syndrome and inflammatory dysfunction can usually be detected in children (Huang et al. 2009). However, onset of the actual metabolic syndrome conditions and associated comorbidities, including inflammation-mediated diseases (e.g., atherosclerosis), may not occur until adulthood. Recognition of the connection between immune dysfunction, low-level chronic inflammation, and metabolic syndrome appears to be growing (DeWitt and Dietert 2010). In fact, obesity itself is sometimes viewed as a chronic, low-grade inflammatory state (Lacasa et al. 2007). For this reason, the functional status of both T-lymphocyte populations and macrophages in the child is an important consideration relative to obesity (Nishimura et al. 2009; Schaffler and Scholmerich 2010). Figure 3 shows the windows of immune maturation that are likely to be most relevant to metabolic syndrome. It also illustrates categories of environmental risk factors that appear to influence the risk of metabolic syndrome (DeWitt and Dietert 2010; Dietert and Dietert 2010).

The probable relationship between immune status and metabolic syndrome is related to the suggestion that adipose tissue functions much like an immune organ. This is not a surprising concept because a variety of specific tissues and organs (i.e., brain, liver, gut, lung, and skin) have been increasingly recognized as having their own specialized microimmune systems, which are critical in determining both pediatric and adult function and dysfunction (Barone et al. 2009; Bienenstock and McDermott 2005; Carpentier and Palmer 2009; Heier et al. 2008; Nemeth et al. 2009; Zaba et al. 2009). Additionally, the close interaction between preadipocytes, adipocytes, and macrophages (resident and infiltrating), the capacity of resident adipose cells to produce immune cytokines, and the capacity of preadipocytes to convert into macrophage-like cells (Isakson et al. 2009; Schaffler et al. 2007) further support the idea that adipose tissue has a major immune function.

Adipose tissue macrophages come in different forms based on cell surface phenotype. Additionally, significant changes occur in adipose tissue in conjunction with elevated risk of metabolic syndrome conditions. For example, in mice, these macrophage subpopulations have been termed M1 and M2. The M1 macrophages tend to produce more tumor necrosis factor-α (TNF-α) and chemokine ligand 2 (CCL2), whereas the M2 macrophages are associated with higher interleukin-10 (anti-inflammatory) cytokine production (Fujisaka et al. 2009). The M1 and M2 ratio appears to be a significant factor in insulin sensitivity (Fujisaka et al. 2009). In adipose tissue, factors secreted by infiltrating macrophages appear to alter preadipocyte differentiation, producing a proinflammatory and fibrotic environment that appears to be critical in the maintenance and progression of fat mass in obesity (Keophiphath et al. 2009).

Several events that are altered in early childhood have been associated with elevated risk of metabolic syndrome. For example, children who mature early (measured by growth parameters) reportedly have a higher risk of young adult metabolic syndrome than do those who mature later (Sun and Schubert 2009). These associations may reflect changes in hormone levels that may affect both body mass index and timing of pubescence. Additionally, a distinct inflammatory profile underlies obesity-related metabolic disorders (Wisse 2004), and a marker of pediatric low-grade inflammation (white blood cell count) has been suggested as a possible marker for metabolic syndrome based on recent reports (Lee et al. 2010).

Immune-inflammatory dysfunction is also seen with prenatal exposures to environmental risk factors that produce postnatal lipid dysregulation. Zelikoff and colleagues demonstrated that both are postnatal outcomes after *in utero* exposure of mice to cigarette smoke. Prenatal exposure to cigarette smoke caused suppression of cytotoxic T-lymphocyte function while also promoting transplanted tumor growth in male offspring (Ng et al. 2006). Cigarette smoke exposure in early life also altered immune homeostasis in offspring (Ng et al. 2008). Similar prenatal exposures to cigarette smoke produced alterations in blood lipids and weight gain in the offspring (Ng et al. 2009). In this latter study, the fat composition of the postnatal diet exerted an influence on the pattern of metabolic effects seen among male or female offspring.

Odegaard et al. (2007) reported that macrophage polarization, including overproduction of CCL2 by macrophages in adipose tissues, is associated with the insulin resistance component of metabolic syndrome. Interestingly, psoriasis cases are reported to have a higher level of proinflammatory cytokines than normal and are comorbid with both lipid abnormalities and metabolic syndrome (Channual et al. 2009; Gottlieb et al. 2008). This particular skin disorder may be associated with elevated production of TNF-α. Additionally, reduced numbers and function of T-regulatory cell populations (CD4^+^Foxp3^+^) occurred concomitantly with insulin resistance (Feuerer et al. 2009). The underlying basis of the connection between inflammatory dysfunction and metabolic syndrome warrants additional investigation.

### Applying the metabolic syndrome example as a general approach

The significance of using the metabolic syndrome pattern to address environmental health risks is that, rather than focusing solely on factors related to stroke versus diabetes versus heart disease, there is an opportunity to design prevention and intervention strategies that can affect not just one disease but an entire associated pattern of chronic conditions. When considering metabolic syndrome, the immune dysfunction component may be causative or simply an associated or disease-facilitating characteristic. Future research will help to address this question. However, for other patterns of environmentally influenced disease, the role of immune dysfunction is clear. In the following examples of “patterns,” the entryway condition or disease is clearly tied to early-life environment, DIT, and pediatric immune dysfunction. A “pattern” approach has already proved integral to the use of metabolic syndrome as a focal point for reducing the risk of cardiovascular disease, diabetes, and stoke. Similar benefits should be obtained by applying a similar “pattern” approach to other entryway diseases and conditions, including those that are based on underlying immune dysfunction.

## Using Immune Dysfunction-Based Patterns of Disease

The identification of prototypical immune-based patterns of disease emerged in a recent sequence of publications (Dietert and Zelikoff 2009, 2010). The authors compared the reported risk associations among 28 diseases and conditions where the entryway disease was prominent in childhood and where early-life environmental risk factors were thought to be related to the induction of the disease (Dietert and Zelikoff 2010). In many cases of cohorts with the primary immune-related conditions, the secondary diseases did not arise until adulthood. Although some disease comorbidities were expected (e.g., the atopic triad of childhood asthma, allergic rhinitis, and atopic dermatitis), others were far less obvious (i.e., various cancers, cardiovascular diseases, neurobehavioral diseases, metabolic disorders, sleep disorders).

### Categorizing patterns of immune-based disease

Four categories of immune-based patterns of disease were previously defined based on the nature of the pediatric entryway disease (i.e., allergic diseases, autoimmune diseases, inflammatory diseases, and recurrent infectious diseases) (Dietert and Zelikoff 2010). Given information presented here, it seems likely that designation of a fifth category, “metabolic diseases,” would be appropriate. It is important to note that secondary diseases and conditions within a given pattern were not in any way restricted to the category of the initial disease. Thus, pediatric allergic disease could give rise to an elevated risk of certain autoimmune, inflammatory, cancerous, and behavioral conditions. Likewise, certain recurrent pediatric infections were associated with an elevated risk of specific allergic, autoimmune, and inflammatory diseases (Dietert and Zelikoff 2010).

There are at least two ways in which these patterns of disease with environmental influences can be used for health risk reduction: to better map the routes to specific diseases whose elevated risks are found in multiple immune-related patterns, and to serve as the basis for environmental protection and therapeutic strategies focused on multiple interconnected diseases rather than single diseases in isolation. These two applications are illustrated in the following sections.

## Using Patterns of Immune-Based Disease for Improved Health Risk Reduction

To consider the utility of patterns of immune-dysfunction–based disease for reducing environmental health risks, any number of entryway diseases could have been selected from our initial analysis (Dietert and Zelikoff 2010). For example, celiac disease presents an interesting pattern because the entryway disease has both allergic and autoimmune components. However, for the present discussion, we focused on a set of examples where entryway diseases represent different categories of immune-based diseases (allergy, autoimmunity, inflammatory, infectious) and are focused in different specialized tissues (lung, skin, brain, gastrointestinal tract, pancreas).

**Figure 4:**
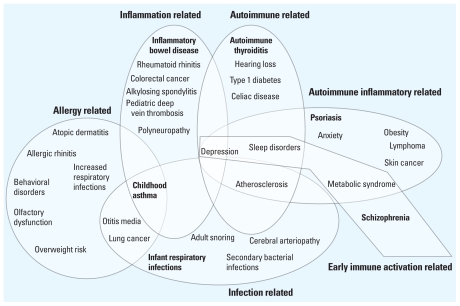
Venn diagram showing six patterns of immune-related diseases and the interactions among the patterns. Designated entryway diseases are shown in bold type within each pattern. Reported comorbid diseases or conditions are included for each pattern and those shared among multiple patterns fall within the intersections of the patterns.

Figure 4 depicts a Venn diagram of six such immune-based disease patterns. This shows the individual patterns. But, more importantly, it shows the intersection of different patterns and the sharing of increased risk for an individual disease by more than one pattern. The six patterns are derived from information presented in Dietert and Zelikoff (2009). In Figure 4, entryway diseases are designated in bold type within each pattern. For each entryway disease, there are prenatal and childhood environmental risk factors that are thought to play a major role in disease development. Among the population diagnosed with the entryway disease, elevated risk of secondary diseases is indicated within each pattern.

The patterns diagrammed in Figure 4 provide several take-home messages, some that reinforce prior observations, and some that open up new opportunities for investigation. Entryway diseases are each connected to several other chronic diseases via elevated risk. Specific underlying immune dysfunction is likely to drive the comorbidities. In Figure 4, several routes appear to result in an increased risk of childhood asthma, with allergic, infectious, and inflammatory components all being important. Depression, sleep, sensory, and psychological disorders are prominent in environmentally influenced immune diseases, and the first three conditions cluster among various autoimmune and inflammatory disease patterns. Earlier-life, immune-based diseases focused in specific tissues (e.g., lung, gastrointestinal tract, skin) are invariably associated with an elevated risk of later-life cancer in that tissue (Dietert, in press).

**Figure 5:**
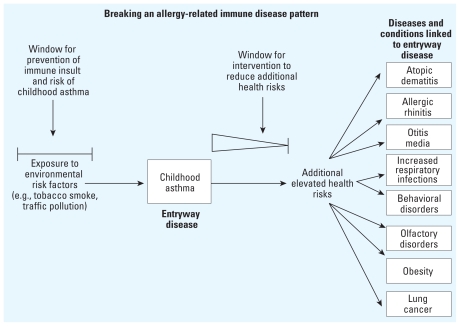
Strategies for breaking a pattern of immune-dysfunction–based disease using childhood asthma as the entryway condition. Diseases and conditions with reported elevated risk among asthmatics are shown on the far right. The first window on the left illustrates the opportunity to prevent the initiation of this disease pattern by blocking DIT-induced immune dysfunction and elevated risk of childhood asthma. The second window shows the opportunity to intervene in the pattern and reduce the risk for progression to associated diseases beyond childhood asthma. Prompt intervention focused on this pattern may be more effective than treatments that are begun just before the onset of additional diseases. The importance of timing of appropriate intervention for likelihood of success in breaking the pattern is depicted using a diminishing triangle. Both the prevention and intervention strategies are important components of overall health risk reduction. Data from Dietert and Zelikoff (2008, 2010).

Figure 5 depicts the pattern for childhood asthma as an entryway disease using a flow chart for enhanced environmental health risk reduction. The diagram illustrates the connection between environmental risk factors and the complete spectrum of potential health risks. Elevated health risks are either well established or strongly suggested (Dietert and Zelikoff 2009, 2010). When the scope of elevated health risks associated with childhood asthma are viewed beyond the single disease, the focus on effective prevention and management of childhood asthma is affected as well.

## Prevention of Immune Insult and Entryway Diseases: Scope and Limitations

For those patterns of disease with immune involvement, preventing the underlying immune dysfunction is the single most effective option to minimize the risk of one or more chronic diseases later in life. Effectively managing the developing immune system includes not only the identification and avoidance of environmental hazards such as immunotoxic chemicals and drugs, but also the optimization of maternal and pediatric diets and reduction of infectious agent triggers of immune-based disease. At the individual level, genetic background and epigenetic effects can also be useful considerations.

Because pediatric immune dysfunction can take many different forms, a focus on safety testing that relies primarily on adult exposure data and directed toward immunosuppression or allergenicity of a test chemical itself is limited, if not misdirected. A disconnect between testing goals and actual DIT-induced health risks was discussed in both a recent review (Dietert 2009b) and a subsequent forum on the topic (Spivey 2009). Many of the entryway diseases of concern today are linked with exaggerated, inappropriate, or misdirected immune and inflammatory responses and are likely to involve disruption of immune maturation during early life. There are several serious implications of this dysfunction–disease connection. Continued reliance on immune safety assessment using adult exposures to accurately predict risk of asthma and type 1 diabetes in children is inappropriate. For this reason, DIT testing should be required for the safety assessment of chemicals and drugs, and the evaluation performed should be directly relevant to the human immune-based diseases of greatest environmental health concern (Dietert 2009b).

Effective prevention of patterns of disease involves implementation of effective strategies to identify environmental risk factors during critical windows of early life and to more effectively protect vulnerable populations from priority risks. In the present review, the focus is on critical windows of immune vulnerability and exposures to environmental risk factors that elevate the chance of pediatric immune dysfunction. The importance of the first topic, risk of DIT, cannot be emphasized enough. If potentially problematic exposures to environmental risk factors that contribute to pediatric immune dysfunction and entryway diseases remain unknown, the disease pattern is likely to be initiated, or alternatively, the immune system may be primed for heightened environmental sensitivity in later life. Once these early immune insults have occurred, the relative effectiveness of intervention strategies may be lessened, and the comparative cost may be increased. Therefore, knowledge of the risk factors producing DIT is critical for making informed decisions for health risk reduction.

At present, the risk factors for pediatric immune dysfunction have not been comprehensively identified. This is not surprising given that DIT testing is not required at present (Dietert 2009b; Spivey 2009). Additionally, even when DIT testing may be performed, the parameters measured can lack relevance for allergic, autoimmune, inflammatory, and metabolic immune-based disease. Clearly confidence in the extent to which risk factors have been identified influences the quality of the decisions made. Improved knowledge of immune risk factors will result in fewer data gaps in risk reduction policies and, therefore, more effective and refined strategies for protecting children against entryway diseases. Obviously, not every risk factor for the developing immune system contributes to the same extent toward entryway diseases. Additionally, some risk factors for immune dysfunction are more easily avoided than are others. For this reason, priorities can be set to address the greatest risks as well as those most easily avoided when it comes to protecting pregnant women and children from exposure.

## Intervention to Disrupt Disease Patterns: Scope and Limitations

Acceptance of the existence of patterns of immune-based disease has inherent ramifications. Among these is the concomitant recognition that, even within an individual, underlying immune dysfunction contributes to elevated risk for not one but several chronic diseases across a lifetime. For this reason, it is advantageous to be able to minimize the extent to which a specific pattern of disease can progress. A diagnosis of childhood asthma has certain implications for present medical care and health management. However, the management of pediatric asthma might be distinctly different if elevated risk of sleep disorders, lung cancer, obesity, recurrent respiratory infections, otitis media, behavioral disorders, and olfactory dysfunction are added to the pediatrician’s concerns for a child’s health. For some of the comorbidities, risk reduction may occur with present management of entryway diseases. But for other comorbidities, special interventions may be needed to break the entryway disease pattern. This is more likely to occur if physicians are aware of the existence of disease patterns and the entryway disease or condition is treated with the health care goal of breaking the overall disease pattern rather than the potentially more restrictive goal of managing the symptoms of the initial presenting disease.

Problems arise when and if current management of childhood asthma, type 1 diabetes, pediatric celiac disease, or recurrent otitis media deals only with the clinical symptoms but fails to address the underlying immune dysfunction that led to the entryway disease. The former treatment course would be more likely to leave intact the elevated risk of one or more additional diseases than would a pattern-based pediatric therapeutic approach (Dietert and Zelikoff 2009). This last point can be highlighted by the potential considerations for managing type 1 diabetes in children. Treatments often focus on lifelong insulin administration, regulation of blood glucose levels, careful dietary controls, and effective exercise (Haller et al. 2005; Hood et al. 2009). But given that this population of children carries an elevated risk for additional autoimmune (autoimmune thyroiditis, celiac disease, multiple sclerosis) and inflammatory conditions (atherosclerosis, depression, sleep disorders) (Dietert and Zelikoff 2009), insulin and glucose management alone is unlikely to correct underlying immune dysfunction. In contrast, therapies that include comprehensive immunomodulatory procedures (Zhao et al. 2009) have the potential to correct the persistent immune defect(s) and reduce the serious additional health risks.

As shown for patterns connected to metabolic syndrome (Figure 2) and childhood asthma (Figure 5), intervention can be used to disrupt a pattern of disease. In theory, this intervention could occur anytime after underlying immune dysfunction is identified. But in practice, this would occur after the diagnosis of the entryway disease (and detection of underlying immune dysfunction). A sliding scale is depicted in the intervention window after the onset and diagnosis of each entryway disease or condition. This is used to indicate that, across a lifetime, the most effective and comprehensive intervention opportunity to break a disease pattern and reduce the risk for all associated diseases is likely to be nearest to the time of entryway disease diagnosis (i.e., early in life). The longer underlying immune dysfunction persists as the individual ages, the more difficult it may be to reduce the risk for the entire spectrum of associated diseases.

For example, childhood asthma is often associated with other allergic conditions, and many of these can arise just before or after the appearance of asthma (Leynaert et al. 2000). Therefore, the window of intervention to effectively block the development of other comorbid allergic diseases in asthmatic children may be quite narrow. Likewise, childhood asthma and obesity are often comorbid in young children (Tai et al. 2009). In contrast, lung cancer is most often diagnosed in older adults (Horner et al. 2009). The window of opportunity to intervene effectively and reduce the risk of lung cancer among asthmatic children is likely to be greater than for reducing the risk of other allergic conditions or childhood obesity. However, even for adult-onset diseases such as lung cancer, as each decade of life passes with continuing immune dysfunction and mounting immune-inflicted insult to the airways, effective intervention is likely to be more difficult.

In general, a global medical approach that includes attention to any underlying immune dysfunction early in life, such as immediately after a pediatric entryway disease is diagnosed, should be useful. It is more likely to break an immune-based pattern of disease than sequential, condition-restricted medical treatment of each condition (resulting in polypharmacy) within a pattern.

## Conclusions

Strategies to break patterns of environmentally influenced illnesses and diseases provide tools to reduce the risk of multiple diseases over a lifetime by using a more holistic preventative and therapeutic approach. Use of patterns of environmentally influenced disease provides novel opportunities for prevention of pediatric- and adult-onset chronic disease, and an opportunity to disrupt patterns of disease through forward-looking therapeutic interventions. As discussed in this review, immune dysfunction appears to be at the center of many patterns of disease, and the pivotal role of immune homeostasis in tissue and organs has probably been underappreciated relative to health risks across a lifetime.

Several recently defined patterns of disease that have been described in this review illustrate how both individual patterns of immune-related disease as well as intersections of multiple disease patterns can be used for reduction of environmental health risks. Although this type of holistic approach to environmental health risk reduction appears promising, it would require several changes away from more traditional approaches. For example, evidence of immunotoxicity after adult exposure should not be a prerequisite for developmental immune safety testing. Instead, DIT testing should be required in the safety assessment of chemicals and drugs and should use end points of direct relevance to the most significant immune-based human diseases. Additionally, therapeutic approaches toward entryway diseases of a pattern should consider the totality of health risks within the pattern rather than focusing solely on the immediate clinical symptoms.
